# High-throughput targeted amplicon screening tool for characterizing intrahost diversity in Staphylococcus aureus directly from sample

**DOI:** 10.1099/mgen.0.001427

**Published:** 2025-06-25

**Authors:** Tara N. Furstenau, Ryann Whealy, Skylar Timm, Alexander Roberts, Sara Maltinsky, Sydney J. Wells, Kylie Drake, Ann Ross, Candice Bolduc, Talima Pearson, Viacheslav Y. Fofanov

**Affiliations:** 1 School of Informatics, Computing, and Cyber Systems, Northern Arizona University, Flagstaff, AZ, USA; 2 Pathogen and Microbiome Institute, Northern Arizona University, Flagstaff, AZ, USA; 3 Mobile Dentistry of Arizona, Mesa, AZ, USA

**Keywords:** genotyping, intrahost single nucleotide variants, population diversity, *Staphylococcus aureus*, targeted amplicon sequencing

## Abstract

A significant proportion of people are asymptomatic carriers of *Staphylococcus aureus* (SA), an important risk factor for the development of opportunistic infections. SA colonization is dynamic, appearing and disappearing, with strains evolving and potentially shifting in composition over time and between body sites. These changes make detection challenging, and the numerous potential sources of reintroduction from other people and even other body site reservoirs preclude efficient efforts to prevent transmission and spread. Identifying typical sources is therefore critical for mitigation. Whole-genome sequencing (WGS), ideally of multiple colonies from multiple body sites, is the gold standard for characterizing SA strains and confirming transmission. However, this is often too resource-intensive for initial assessments of transmission and not feasible for large-scale studies involving various body sites from multiple individuals over time. To address these challenges, we developed a low-cost, custom, species-specific amplicon sequencing (AmpSeq) assay optimized to provide high-resolution discrimination of SA genotypes directly from samples. We tested this approach on a subset of samples that were a part of a large-scale longitudinal study of SA carriage. Oral and nasal samples were collected from nine participants every 2 weeks for up to 18 weeks and qPCR positive samples were analysed using our AmpSeq assay directly from the sample without culturing. The longitudinal sampling strategy enabled us to characterize changes in SA colonization patterns over time, detect potential strain mixtures and identify rare variants that may serve as signatures of transmission between different body sites or among individuals. Without using WGS, we were able to rapidly eliminate the possibility of transmission between sampled residents. Participants who had positive oral and nasal samples had no fixed SNP differences between the two body sites, suggesting likely within-person spread. Analysis of rare variants segregating in the oral and nasal populations suggests that the nasal populations were the likely source of the spread because the nasal samples had higher diversity and most of the variants identified in the oral samples were shared with the nasal samples. While WGS can be used to provide higher resolution to colonization patterns and validate these findings, our AmpSeq approach offers a rapid, cost-effective, direct-from-sample method for species-specific screening intended for population-level characterization that allows researchers to strain type, identify or eliminate likely transmission cases and identify potential reservoirs before resorting to more expensive WGS methods.

**Impact Statement**

Colonizing opportunistic pathogens like *Staphylococcus aureus* present a unique challenge for disease study because rather than causing acute infections upon transmission, they persist asymptomatically for long periods of time, allowing the bacterial population to evolve and differentiate. Characterizing the diversity within these populations is important for choosing correct treatments, quantifying the risk of horizontal gene transfer and understanding paths of transmission between people and spread to different body sites. The gold-standard approach for characterizing population diversity is through culturing and whole-genome sequencing of multiple colonies per sample, which is labour-intensive and expensive for any large-scale study. Using a custom-designed species-specific amplicon sequencing assay, we offer a cost-effective method for characterizing the diversity in *S. aureus* populations directly from samples without the need for labour-intensive culturing or whole-genome sequencing. Our small-scale study highlights how this method provides a scalable tool for large epidemiological studies ideal for systematically exploring broader patterns of carriage and transmission.

## Data Summary

The authors confirm all supporting data have been provided within the article or through supplementary data files. The sequencing reads have been deposited in NCBI’s Sequence Read Archive (BioProjects: PRJNA1166327, PRJNA1209594 and PRJNA1209589), and the accession numbers are provided in Tables S4, S11 and S12, available in the online version of this article.

## Introduction

*Staphylococcus aureus* (SA) is a significant public health concern due to its ability to cause a wide range of infections, from mild skin infections to life-threatening conditions such as bacteraemia, pneumonia and endocarditis [1,3]. The emergence of antibiotic-resistant strains, such as methicillin-resistant SA (MRSA), has further heightened the risk associated with SA, complicating treatment options and leading to higher morbidity and mortality rates [4,6].

SA lives in close association with humans and can persist asymptomatically for long periods of time. Various body sites are frequently colonized, particularly the skin and mucosal surfaces, and opportunistic infections occur when the bacteria penetrate these barriers [7,10]. While infections can be due to extrinsic sources, there is a tight link between carriage and self-infection [2,7, 10,15]. Much remains unknown about carriage patterns, and although it is typically reported that ~33% of the population are *S. aureus* carriers in their nose (~2% for MRSA), as detection methods improve and multiple body sites are tested, these numbers can increase to >65% [6,16]. Carriage is most often identified in the anterior nares, but it is often found in the oropharynx, intestines, skin and other mucosal surfaces [10,17, 18]. SA colonized body sites can serve as reservoirs for spread to other body sites, endogenous infections and transmission to others [11,19, 20]. Longitudinal studies assessing changes in presence/absence, pathogen quantities and genotypes can produce valuable insights into carriage but have been severely limited due to a lack of high-throughput and high-resolution methodologies [19,21,28].

Understanding the dynamics of bacterial populations within hosts is crucial for understanding carriage and transmission patterns. Compared to the short timeframe of an acute infection, the persistence of SA during long-term carriage allows for the accumulation of mutations that can be lost or propagated through genetic drift or selection [29,31]. Additionally, co-colonization of multiple strains occurs in up to 21% of colonized individuals [32,35] and can increase the potential for horizontal gene transfer and promote the spread of antibiotic resistance and virulence genes [36,38]. Failure to capture intrahost diversity can complicate the diagnosis, treatment and tracking of infections, as different strains within the same host may respond differently to antibiotics or evade immune detection. Characterizing pathogen genetic diversity can also help in identifying key reservoirs and tracing transmission pathways [22,39] that may be missing or incomplete if only a small number of representative isolates are compared. By comprehensively studying intrahost diversity, we can better understand the evolution of SA within hosts, improve infection control strategies and develop more effective interventions to prevent the spread of this pathogen.

Whole-genome sequencing (WGS) is currently the gold-standard method for characterizing SA strains because it provides comprehensive genomic information. However, accurately capturing the full complexity and heterogeneity of a colonizing SA population requires sequencing multiple isolates from each sample, as single isolates are not representative of the entire population’s diversity [33,40]. Additionally, sampling from multiple body sites is necessary to identify all potential reservoirs of SA within a host, while sampling multiple time points is necessary for understanding how the colonizing population changes or evolves over time. WGS approaches provide invaluable insights but also present significant challenges. The large number of samples needed to systematically characterize longitudinal within-host diversity and the necessity of culturing, isolating and sequencing numerous colonies from each sample make WGS approaches both time-consuming and cost prohibitive.

PCR-based molecular typing methods like Multilocus Sequence Typing (MLST) [41] that are adapted to a next-generation sequencing platform offer a more economical alternative to characterizing population variation. PCR-based approaches can be applied directly to the sample without the need for culturing and can provide very high coverage of population variation within the targeted regions even with low concentrations of starting DNA. Unfortunately, the small number of housekeeping genes targeted by traditional MLST provides a comparatively low discriminatory resolution that is not optimal for either confirming or refuting transmission events with any degree of confidence, and the genes/primers are often not species-specific, making it difficult to use for population-level characterization. However, if a larger number of strategically selected targets were queried, targeted amplicon sequencing (AmpSeq) could offer a cost-effective way to genotype the sample and characterize the diversity within bacterial populations.

We are currently conducting a large-scale longitudinal study of SA carriage and transmission that involves the collection of oral and nasal swabs every 2 weeks. Our goals are to (1) characterize SA carriage rates, (2) identify circulating strain types, (3) track how colonizing strains are replaced or evolve over time, (4) characterize the frequency and dynamics of co-carriage of multiple strains (within or between body sites), (5) determine if there are associations between strain types and body sites, (6) determine which body sites are more likely to act as reservoirs for the spread of SA and (7) detect possible transmission between individuals. Answering these questions using WGS on tens of thousands of isolates (assuming multiple isolates per positive sample) is costly and inefficient. Therefore, we need a rapid, high-throughput, cost-effective method to characterize the colonizing population and help screen for likely reservoirs and transmission pairs so that WGS resources can be reserved for validating and providing additional population genetic details for only these candidates. Here we describe a custom AmpSeq assay designed to address these challenges.

## Methods

### Development of multiplexed targeted AmpSeq assay

Our custom AmpSeq assay was designed to target genomic sites that are conserved across strains but contain polymorphisms that maximize strain differentiation within SA. The sites were bioinformatically confirmed to be species-specific so that similar loci in other common commensal *Staphylococcus* species will not amplify and confound variant detection.

A set of 961 SA reference genomes was downloaded from the RefSeq database [42] using the ncbi-genome-download tool (https://github.com/kblin/ncbi-genome-download). These genomes included all complete assemblies deposited in the database up to the year 2022. The genomes were aligned to the NCTC 8325 reference genome (NC_007795.1) using NUCmer v3.1 [43] and SNPs were identified using NASP v1.2.1 [44] with default parameters after masking duplicated loci. Regions of high similarity with 12 other *Staphylococcus* species commonly found in human samples (*Staphylococcus epidermidis*, *Staphylococcus haemolyticus*, *Staphylococcus saprophyticus*, *Staphylococcus hominis*, *Staphylococcus warneri*, *Staphylococcus capitis*, *Staphylococcus simulans*, *Staphylococcus cohnii*, *Staphylococcus xylosus*, *Staphylococcus saccharolyticus* and *Staphylococcus lugdunensis* [45,47]) were also masked. These regions were identified by aligning reference genomes from each of these species to the SA reference using NUCmer and removing any regions where alignment was successful. The reference sequence accessions and the regions masked due to alignment with other species are provided in Tables S1 and S2, available in the online Supplementary Material.

VaST [48] was used to identify a minimal set of target loci that maximizes differentiation between the genomes. This is a greedy optimization tool that iteratively adds loci (groupings of SNPs in non-overlapping 100 bp windows) that provide the most improvement in entropy-based diversity among the provided genomes. The first 27 targets were selected because additional targets did not substantially improve resolution. Primers (Table S3) were designed to amplify the targets in regions that were highly conserved among the reference genomes, and the primers were optimized to work together in a single multiplex PCR (i.e. minimizing the number of primer interactions and ensuring similar melting temperatures). The primers were synthesized with forward (UT1 : 5′-ACCCAACTGAATGGAGC-3′) and reverse (UT2 : 5′-ACGCACTTGACTTGTCTTC-3′) universal tails to facilitate index ligation prior to pooling for multiplexed sequencing.

### *In silico* phylogenetic comparison of AmpSeq target regions and core genome SNPs

We used reference genomes to assess the concordance between phylogenies inferred from our 27 target AmpSeq assay, an existing MLST scheme and core genome SNPs. To reduce computation time and simplify visualization, we deduplicated the 961 reference genomes using Assembly Dereplicator v0.3.2 [49], retaining 128 unique genomes with a distance threshold of 0.001. After filtering genomes that did not pass IQTree’s [50] nucleotide composition quality control check, 119 genomes were used in the analysis (Table S1). The reference sequences were aligned as described above using Nucmer, and recombinant regions were detected and filtered using Gubbins v3.3.3 [51]. Three maximum-likelihood phylogenies were inferred with IQTree v2.4.0 [50] using (1) concatenated core genome SNPs with substitution model GTR+F+ASC+G4, (2) concatenated sequences from the 27 amplicon target regions extracted from the reference genome alignment (all were in recombination-free regions) using the best-fit substitution model HKY+F+R4 and (3) concatenated sequences from the 7 housekeeping genes targeted in the SA MLST scheme using the best-fit substitution model TPM3u+F+R3 [41]. Branch support was assessed using ultrafast bootstrap approximation (UFBoot) with 1,000 replicates [52]. Robinson–Foulds (RF), matching pairs and quartet distances between the trees were calculated using TreeCmp [53]. A tanglegram was drawn to link the taxa between the trees using the dendextend v1.19.0 [54] R package; the untangle_step_rotate_2side function was used to rotate branches to minimize crossings.

### Empirical comparison of AmpSeq and WGS results

To compare the results of the AmpSeq assay to WGS, we processed DNA extracted from samples previously collected from a study of community carriage in Yuma, AZ (Project 1116783 that was approved by the Northern Arizona University Institutional Review Board) [16,55,58]. Samples were collected from nares using a double-tipped BBLCultureSwab. The swabs were streaked onto CHROMagar *S. aureus* media and incubated for 24 h at 37 °C. All colonies in the pink to mauve colour range were collected and pooled for DNA extraction using Qiagen DNeasy blood and tissue kits (in some cases, a single colony was also isolated). The extracted DNA was prepared for WGS (as previously reported [58]) and AmpSeq (see Amplicon Sequencing, below) on an Illumina NextSeq instrument.

Primer sequences were removed from both the forward and reverse AmpSeq reads using Cutadapt v4.9 [59]. Quality control was performed using Trimmomatic v2.9.1 [60] with the following parameters: LEADING=3, TRAILING=3, SLIDINGWINDOW=4:15 and MINLEN=36. Reads were aligned to the *S. aureus* reference genome (NC_007795.1) using BWA-MEM v2.4.0 [61,62]. Consensus calls were made using BCFtools v1.15.1 [63] with a base quality cutoff of 20, a mapping quality cutoff of 20, a minimum coverage of 10× and a majority (>70%) of reads required to support an SNP call. Only alignments within the expected amplicons, excluding primer regions, were included in the AmpSeq analysis. For the WGS data, only SNPs in non-recombinant regions were included. The concatenated sequences from both approaches were combined with the reference database sequences, and maximum-likelihood phylogenies were inferred using IQTree as described above.

### Longitudinal sample collection

Samples were collected every 2 weeks from residents of three long-term care facilities in the Phoenix metropolitan area. Informed written consent was received from each participant, and the project was approved by the Northern Arizona University Institutional Review Board (Project 1766728–3). Swabs of the anterior nares and mouth were self-administered under the supervision of study staff to ensure consistency. This method has been highly successful in detecting SA in previous studies [16,55, 56]. After sampling, swabs were stored in 1 ml of Liquid Amies medium in a −20 °C freezer before processing.

### DNA extraction and *S. aureus* detection

DNA was extracted from the collection media using an Applied Biosystems MagMax DNA Multi-Sample Ultra 2.0 extraction kit on a KingFisher Flex (Thermo Fisher) instrument. The SaQuant qPCR assay [16,64] was run in 10 µl reactions using 5 µl of Applied Biosystems TaqMan Universal PCR MasterMix, 1 µM of forward primer, 1 µM of reverse primer, 200 nM of TaqMan TAMRA probe and 1 µl of template. Thermocycling conditions were as follows: hot start TaqMan activation (10 min at 95 °C), followed by 40 cycles of denaturation (15 s at 95 °C) and extension (1 min at 57 °C). Serial dilutions of quantitative genomic DNA were included to allow for DNA quantification using standard curve analysis (Ct and DNA quantities are available in Table S4).

### Amplicon sequencing

The KAPA 2G Fast Multiplex PCR Master Mix was used with 27 primer pairs at 0.2 µM concentration, with 5 ng of template DNA and enough water to bring the reaction mixture to 25 µl. The PCR thermocycling conditions consisted of an initial denaturation at 95 °C for 3 min, followed by 35 cycles of denaturation at 95 °C for 15 s, annealing at 60 °C for 30 s and extension at 72 °C for 1 min and 30 s and a final extension at 72 °C for 1 min. The PCR products were then prepared for sequencing by performing a bead clean-up using a 2:1 ratio of AMPure beads (Beckman Coulter) to the samples. Following clean-up, an extension PCR was performed to add indexing barcodes with the following cycle profile: 98 °C for 2 min, six cycles of (98 °C for 30 s, 60 °C for 20 s and 72 °C for 30 s), 72 °C for 5 min and 10 °C indefinitely. A second bead clean-up was performed with the same specifications, and libraries were quantified for pooling using a KAPA quantification kit. Samples were sequenced on an Illumina MiSeq platform to generate 150 bp paired-end reads.

### SNP calling and rare variants

The primer sequences were removed from both the forward and reverse sequencing reads using Cutadapt v4.9 [59]. The reads were then processed using Trimmomatic v2.9.1 [60] with the following parameters for quality control: LEADING=3, TRAILING=3, SLIDINGWINDOW=4:15 and MINLEN=36. Reads were aligned to the reference sequence (NC_007795.1) using BWA-MEM v2.4.0 [61,62]. SNPs and rare variants were called using LoFreq v2.1.5 [65] after running the ‘viterbi’ command to correct alignment errors. Consensus SNPs were called if there were at least ten reads covering the site and the majority (>70%) of reads supported the call.

A group of high-confidence rare variants was generated by collecting loci where LoFreq called a low-frequency variant (<2%) in at least one sample. For samples where a rare variant was not called at a position with LoFreq, the missing data were filled with pileup allele frequencies generated using BCFtools v1.15.1 [63] with a base quality cutoff of 20, a mapping quality cutoff of 20 and a minimum coverage of 10× coverage. Only genome ranges within each amplicon, excluding the primer regions, were included in analyses. Because some rare variants were likely a result of errors introduced through PCR and sequencing, we focused on counting rare variants that were detected in samples at multiple time points to increase our confidence that true variants were detected. Rare variant allele frequencies were used to calculate Nei’s genetic distance [66] between the populations at different time points.

### Phylogenetic analysis of samples

To identify the sequence types of the samples, we drew a minimum spanning tree using consensus SNPs from our target sites in both the samples and reference genomes using Grapetree v2.1 [67] using the MSTreeV2 method. The sequence types for the reference genomes were estimated using mlst v2.23.0 [41,68, 69], and sample sequence types were assigned based on the sequence type of the references that they were closest to. To visualize the number of SNP differences between the samples, PAUP v4.0a [70] was used to draw a maximum parsimony tree from the consensus SNPs.

### Mixtures to simulate co-colonization

To test the AmpSeq assay’s ability to detect co-colonization, we created mixtures of *S. aureus* populations collected from different individuals at known proportions. The swabs were streaked onto CHROMagar *S. aureus* media and incubated for 24 h at 37 °C. All colonies in the pink to mauve colour range were collected and combined for DNA extraction as described above. Mixtures of the quantified DNA were made at ~7:3 ratios prior to AmpSeq. The combinations of samples were designed so that the detection of a mixture varied in difficulty from easy (many SNPs between the two samples) to hard (only a few SNPs between the samples). Both the DNA from the individual populations and the population mixtures were amplified and sequenced as described above.

## Results

### Comparison of phylogenies inferred from core genome SNPs and AmpSeq targets

**Fig. 1.**
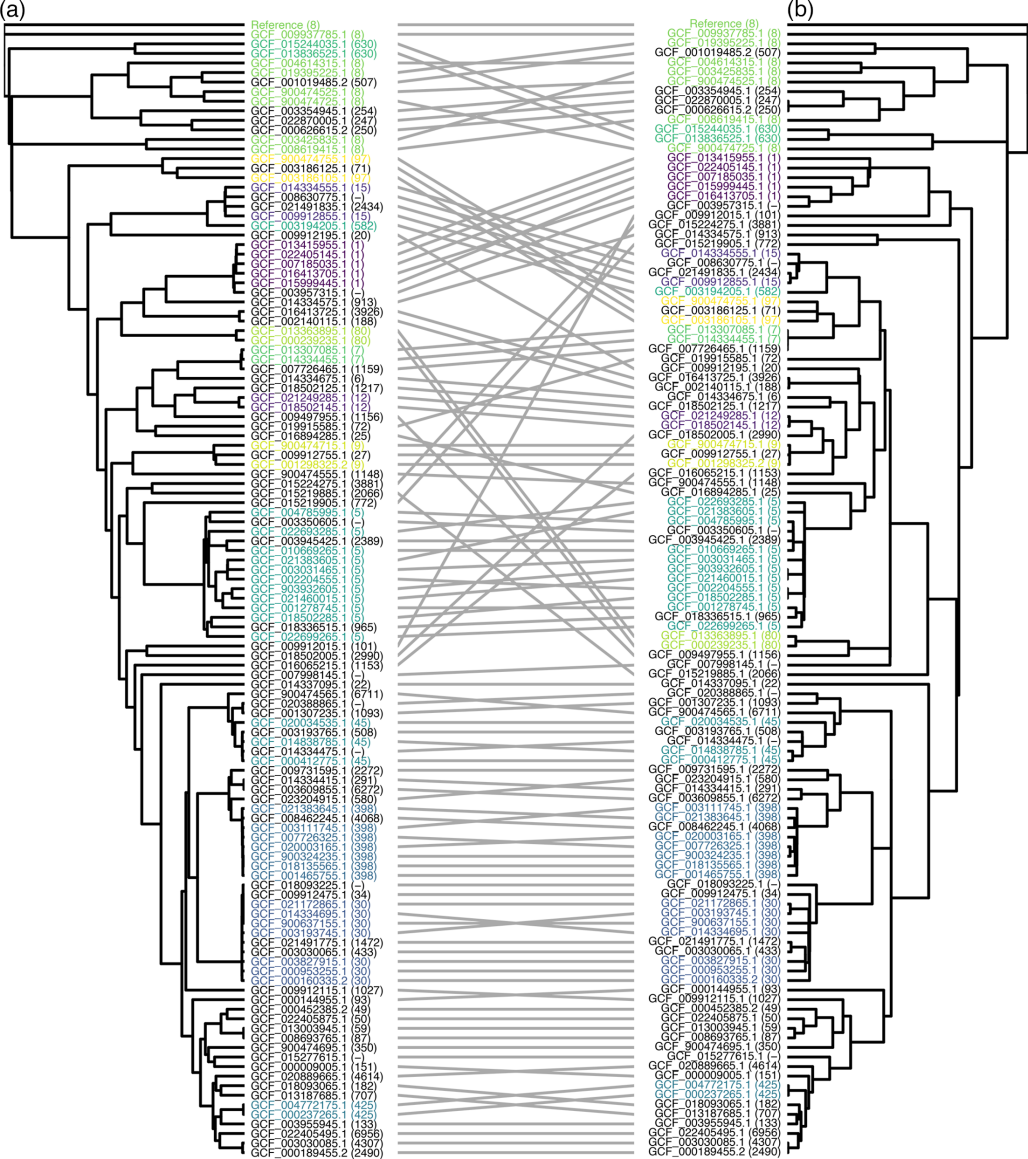
Comparison of phylogenetic trees inferred from (a) core genome SNPs and (b) sequences from 27 amplicons in the AmpSeq assay for 119 *S. aureus* reference genomes. In the tanglegram, lines connect the same taxa across trees, with the degree of crossing indicating topological discordance. Taxa labels correspond to assembly accessions, with the sequence types in parentheses (untyped sequences are denoted by a dash; sequence types that appear more than once are colour-coded).

To evaluate the concordance between phylogenies inferred from WGS and the AmpSeq assay, we compared maximum-likelihood trees constructed from core genome SNPs (Fig. 1a) and sequences from the 27 targeted amplicons (Fig. 1b) extracted from 119 *S. aureus* reference genomes. A tanglegram was used to link the same taxa across the trees and visualize topological discordance. The core genome phylogeny was based on 185,331 total SNPs, including 109,757 parsimony informative SNPs (Table S5). In contrast, the AmpSeq tree was constructed from 3,476 total genome positions, of which 418 were SNPs and 283 were parsimony informative (Table S6). Despite the substantial difference in genomic coverage, the major phylogenetic groups observed in the core genome SNP phylogeny were largely preserved in the AmpSeq tree, and sequence types remained clustered together. While some rearrangements were observed, the broad structure of the phylogeny was maintained, suggesting that the AmpSeq assay captures sufficient phylogenetic signal to distinguish major lineages.

The AmpSeq tree showed greater concordance with the core genome SNP tree compared to the MLST tree (not shown). The MLST scheme covered 3,186 total genome positions, with 210 SNPs and 129 parsimony informative sites, resulting in lower resolution than the AmpSeq targets. The RF distance, which quantifies differences in branching patterns, was lower for the AmpSeq tree (RF=79) than for the MLST tree (RF=89), indicating that the AmpSeq tree more closely resembled the core genome SNP phylogeny. Similarly, the matching pairs distance and the quartet distance, which measure the number of groups of two or four taxa that resolve differently between the trees, were lower for the AmpSeq tree (3,174 and 1,920,914, respectively) than for the MLST tree (4,115 and 2,271,536, respectively).

To further compare the AmpSeq assay to WGS, we conducted an empirical test by sequencing a set of 17 samples using both approaches in parallel. We compared the placement of these samples within the maximum-likelihood phylogenies constructed from the core genome SNPs and the AmpSeq targets. As shown in Fig. S1, the samples occupied similar positions among the reference genomes in both trees. In the AmpSeq tree (Fig. S1B), most branches were well supported, with 50% of branches having bootstrap values of 79.5% or higher.

### AmpSeq of longitudinal samples from two body sites

**Fig. 2.**
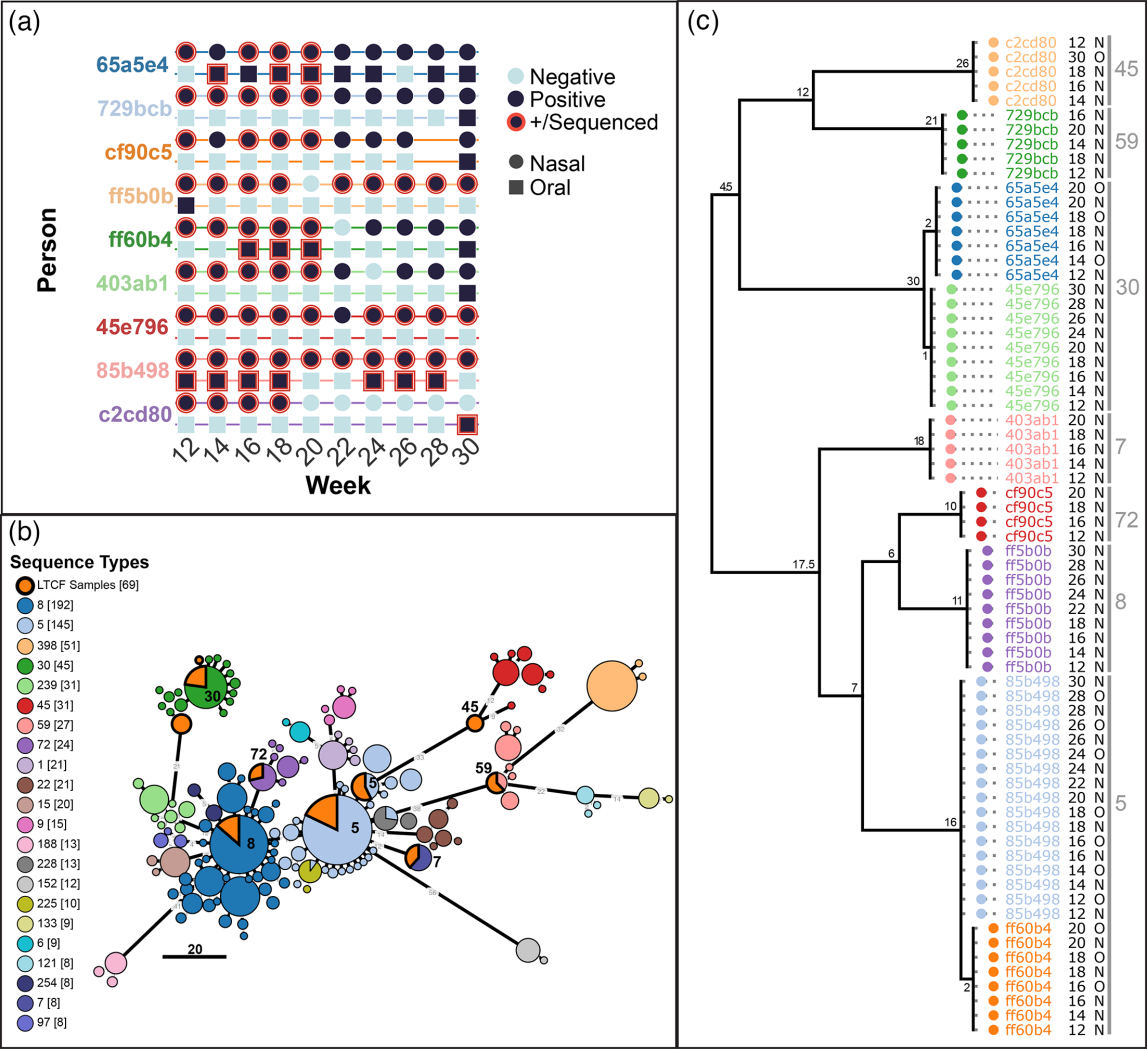
Genotyping SA strains from a longitudinal carriage study using AmpSeq assay. (a) Longitudinal carriage patterns from nine long-term care facility residents. Nasal (circles) and oral (squares) samples were collected every 2 weeks and tested for SA using qPCR. Positive samples are shown in dark blue, and those analysed with the AmpSeq assay are outlined in red. (b) Strain differentiation using AmpSeq targets. A minimum spanning tree was constructed from the targeted amplicon sequences extracted from reference genomes with known sequence types (colour-coded) and AmpSeq results from the study samples (shown in orange). (c) A parsimony tree inferred from consensus SNPs identified from AmpSeq assay. Each sample is labelled with the participant ID, sample week, body site and predicted sequence type. The branch lengths indicate the number of SNP differences between the samples.

To evaluate the performance of the AmpSeq assay for strain characterization, we tested it on a subset of samples from a large-scale longitudinal study of SA carriage in long-term care facilities. We selected samples from residents who had multiple consecutive SA positive samples (via qPCR), including four individuals who were colonized at both the oral and nasal sites. This allowed us to assess both longitudinal carriage and site-specific variation. The positive samples selected for AmpSeq are indicated in Fig. 2(a). For three participants (ff5b0b, 45e796 and 85b498), the sequenced samples spanned 18 weeks (Fig. 2a). Overall, 69 samples were sequenced; 55 were nasal and 14 were oral samples. The median amplicon size excluding the primers was 126 bp (min=66 bp, max=218 bp), and the total length of the amplicons was 3,476 bp (Table S3). The average depth of coverage per position was 716× (sd=492) across all samples, with a minimum of 65× and a maximum of 2,847×. The coverage for the nasal samples was generally higher (mean=827, sd=480) than for the oral samples (mean=279, sd=232). Of the 27 amplicon targets, 22 had consistent coverage with at least 50/69 samples having at least 50× coverage and a minimum average coverage of 250× per locus (Fig. S2). The sample metadata, average coverage, Ct and DNA quantities are available in Table S4.

### AmpSeq strain-level diversity among colonized individuals

The AmpSeq assay revealed substantial genetic diversity among SA strains colonizing long-term care facility residents, and it provided higher resolution for strain differentiation compared to MLST. Fig. 2(b) shows a minimum spanning tree constructed from the targeted amplicon sequences extracted from reference genomes with known sequence types and the AmpSeq results from the study samples. Among the reference sequences, the separation observed between strains with the same sequence type shows that the AmpSeq assay provides finer resolution for distinguishing closely related strains. From the long-term care facility samples, we identified 134 consensus SNPs across 20 amplicons (Table S7). The placement of these samples among the reference sequences shows high levels of genetic diversity for the colonizing strains, with nine distinct genotypes representing seven different sequence types. Within individuals, all longitudinal samples at both body sites were genetically identical at the consensus SNP level for the targeted amplicons. Fig. 2(c) shows a maximum parsimony tree constructed from 133 parsimony informative SNPs with a consistency index (excluding uninformative characters) of 0.74. Although two individuals carried ST30 and two carried ST5, the AmpSeq genotypes were differentiated by at least two SNPs, ruling out direct transmission. All other individuals carried unique genotypes that formed more distantly related monophyletic groups.

### Low-frequency variants maintained in populations over time

**Fig. 3.**
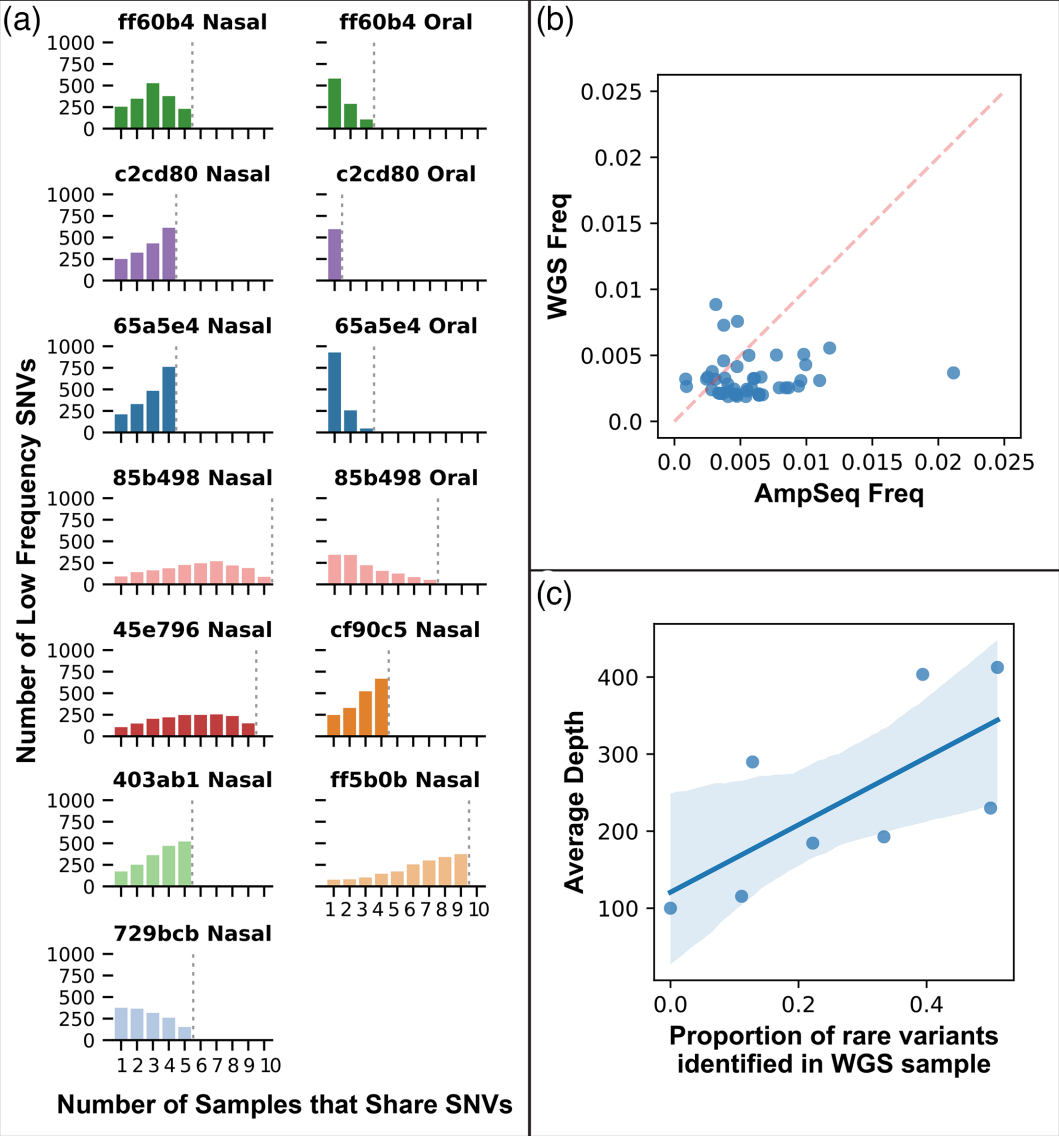
The same rare SNVs were detected within individuals across all time points. (a) The figure shows the number of SNVs that were shared by a given number of samples from the same individual (*x*-axis), separated by body site where applicable. The dashed grey line indicates the total number of samples analysed per individual and body site. The height of the bar on the far right of each plot indicates the number of SNVs that were identified in all samples. (b) Validation of SNVs using parallel AmpSeq and pooled population WGS. The plot shows the frequency of SNVs identified using AmpSeq that were also detected in WGS of the same DNA extraction. (c) The proportion of SNVs confirmed with WGS in each sample compared to the average sequencing depth at each site.

After establishing that the consensus genotypes remained stable within individuals over time, we aimed to determine whether low-frequency variants were maintained within the populations. With deep sequencing of the amplicons, we identified low-frequency (<2%) single nucleotide variants (SNVs) at 1,806 genomic positions (average of 992 per sample) distributed across the 22 amplicons that were consistently amplified (Table S8). These represent point mutations that are segregating in the population that have not yet been fixed or removed. While some SNVs were lost over time or undetected, many were maintained in each population throughout the duration of sample collection (Fig. 3a). The time between samples was not a significant predictor of genetic distance (linear regression, *P*=0.206), while average coverage was significant (*P*<0.001). This suggests that SNVs were more likely undetected rather than lost from the population, further supporting the longevity of variants in the populations. Higher coverage in the nasal samples allowed us to detect SNVs at lower frequencies in the nasal samples (mean=0.0029, sd=0.0016) compared to the oral samples (mean=0.0041, sd=0.0024).

To validate that the SNVs detected by the AmpSeq assay were not artefacts of the method, we performed parallel AmpSeq and WGS on DNA extracted from SA populations (pooled colonies) from eight samples. Our analysis confirmed that, on average, 35% (48/138) of SNVs detected by AmpSeq were also detected with WGS at positions where both methods had coverage >100× (Table S9). The average depth of coverage for these loci was 574× (sd=368) for the AmpSeq samples and 241× (sd=119) for the WGS samples, indicating that lower coverage may have contributed to the lack of detection in some cases. Fig. 3(b) shows the frequencies for the variants identified in both preparations, and Fig. 3(c) shows the relationship between WGS coverage and the percent of rare variants detected in each sample. These results confirm that at least some of the rare variants detected by AmpSeq were also detected by whole-genome population sequencing, and therefore they are likely to be present in the population rather than amplification artefacts.

### Comparing diversity between nasal and oral samples

To better understand the relationship between the nasal and oral SA populations, we examined the relative amounts of diversity at each body site and the number of SNVs shared between these populations. On average, the quantitative polymerase chain reaction (qPCR) results showed that nasal samples had consistently higher DNA copy number estimates and lower Ct values than the oral samples (mean Ct=23 for nasal vs. 30 for oral, Table S4). AmpSeq results also showed higher depth of coverage in nasal samples compared to the oral samples. While these values do not provide a direct measurement of total SA bacterial load, the consistently lower yield from oral samples suggests that the SA burden in the oral cavity may be lower than in the nasal cavity.

**Fig. 4.**
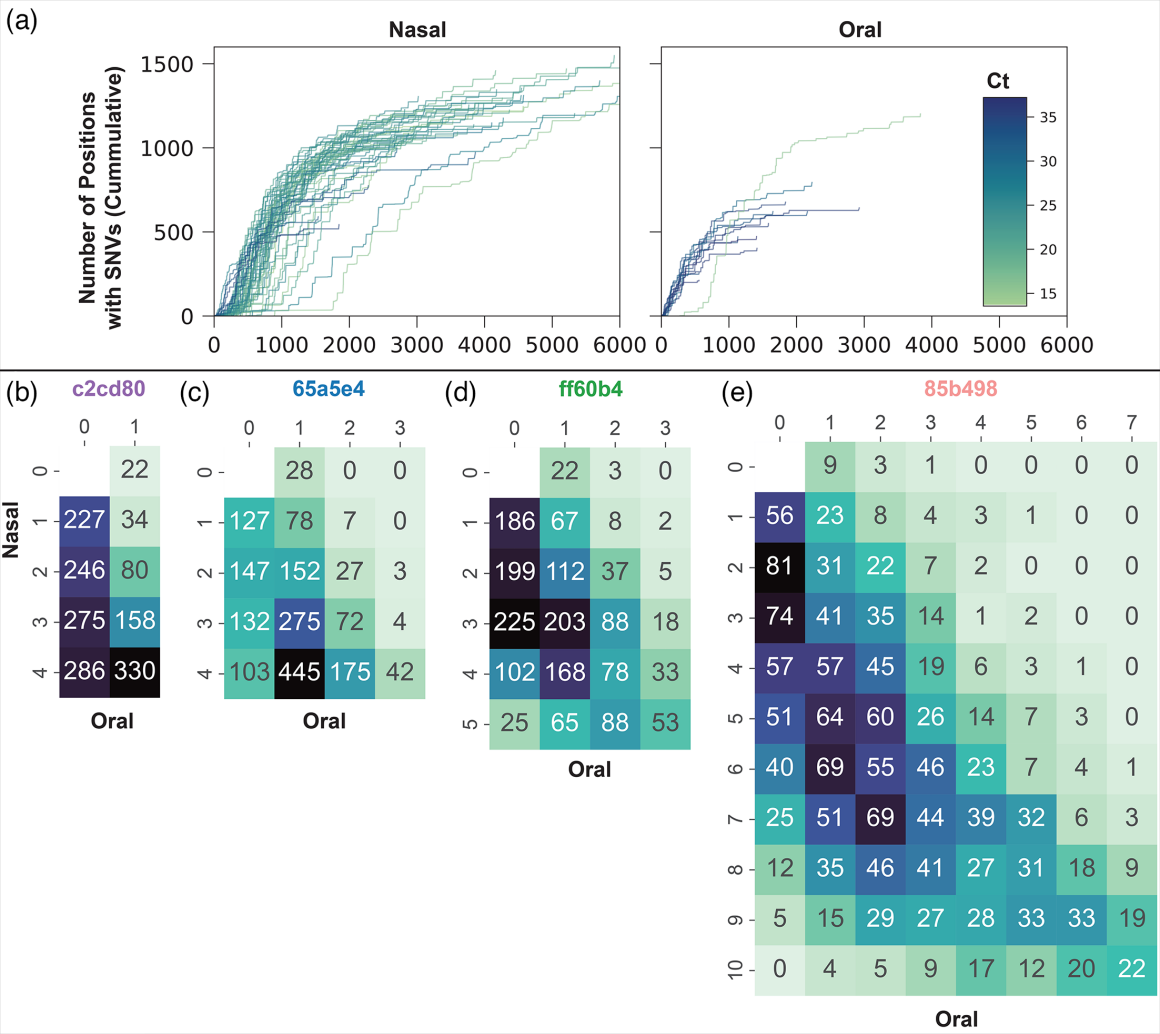
Nasal populations exhibit greater diversity and are likely the source of spread to the oral populations. (a) DNA quantity and the accumulation of SNVs with coverage depth suggest that the effective population size of the nasal samples is larger than the oral samples. The oral samples generally showed higher Ct values than the nasal samples, and the accumulation of SNVs with increasing coverage plateaued more rapidly, suggesting that most of the diversity was captured. (b–e) The heatmaps show the number of times the same SNV was identified among a given number of oral samples (*x*-axis) and a given number of nasal samples (*y*-axis) from each of the four participants. Columns labelled ‘0’ indicate the number of SNVs shared among a given number of nasal samples but not with any oral samples, whereas rows labelled ‘0’ indicate the number of rare variants shared among a given number of oral samples but not with any nasal samples. Few SNVs were shared among multiple oral samples without also being present in the nasal samples. The reverse is not true for the nasal samples, which share many SNVs that were not detected in the oral samples. For (b)–(e), the total number of SNVs represented in each plot is as follows: 1,658, 1,817, 1,787 and 1,842. In some cases, the total number of SNVs is higher than the total number of variable positions (1,806) because samples have different alleles at some positions.

The accumulation of the number of SNVs with increasing depth of coverage plateaued more rapidly in the oral populations than the nasal populations, indicating that most of the genetic diversity was captured with the achieved depth (Fig. 4a). At a given depth, more SNVs were detected in the nasal populations, supporting the idea that the nasal environment harbours a more diverse population. Fig. 4(b)–(e) shows that only a few SNVs were shared among multiple oral samples that were not also shared with the nasal samples. The reverse, however, is not true for the nasal samples, which share many SNVs that were not shared in the oral samples. Combined, this evidence suggests that the nasal populations are more established and are likely to be the source of the oral populations.

For individuals 65a5e4 and ff60b4, the nasal populations were established at least 2 and 4 weeks before oral colonization, respectively (Fig. 2a). Based on the number of SNVs that the oral samples share with the nasal samples (Fig. 4c, d), it seems likely that the nasal population was the source for colonization of the mouth, with frequent introductions. For individual 85b498, four oral samples were positive, followed by two negative samples in weeks 20 and 22, before colonization was re-established for three more timepoints. The source of the re-established oral population could have been either the nasal reservoir, which persisted through weeks 20 and 22, or a severely reduced oral population that persisted below the limit of detection. Our results suggest that the former is more likely because, if a bottleneck occurred as suggested by the latter, we would not expect many variants to be shared with as many of the nasal samples, and we would expect more variants shared among the oral samples (Fig. 4e). Individual c2cd80 had positive nasal samples from week 12 to week 18, then oral and nasal samples were negative until a positive oral sample was detected at week 30. The one oral sample had only 22 (3.5%) low-frequency variants that were not identified previously in the nasal samples and 330 (53%) that were shared with all four of the earlier nasal samples (Fig. 4b). This pattern suggests that recolonization of the mouth originated from (1) a nasal population that persisted below the detection threshold, (2) an unsampled body site or (3) retransmission from an external source that was also the source of the initial nasal population. Nonetheless, the large number of SNVs shared between all the nasal samples and the oral sample is suggestive of a nasal source, and the timing of the positive oral samples suggests that the oral populations likely receive frequent introductions from nasal populations.

### Detecting co-carriage in simulated mixtures

**Fig. 5.**
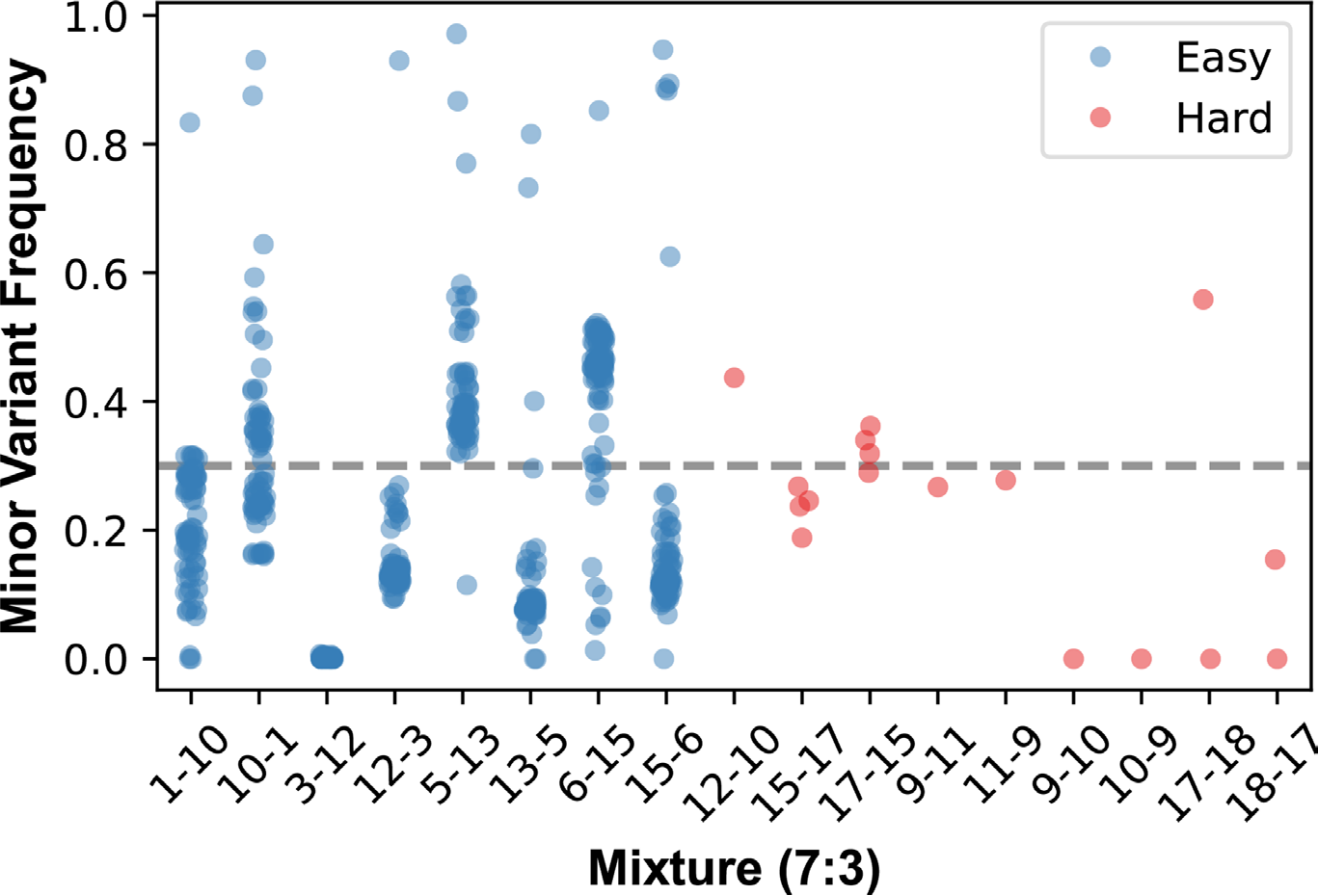
The AmpSeq assay can detect signals of co-carriage of multiple *S. aureus* genotypes in simulated sample mixtures. DNA extracted from *S. aureus* populations collected from different individuals was combined at 7:3 ratios, and the frequency of alleles at sites of expected polymorphism (based on the sequences of the component samples) was detected using our AmpSeq assay. The figure shows the minor allele frequencies for the expected variants for each of the mixtures. Easy mixtures had many (mean=79, sd=1.16) expected variants between the genotypes, and the hard mixtures had between 1 and 4 variants. The dashed grey line indicates the expected minor allele frequency given the proportions of DNA added to the mixtures. The *x*-axis labels indicate the sample IDs that were included in each mixture in major–minor proportion order.

To assess the ability of the AmpSeq assay to detect co-carriage of multiple SA strains within a sample, we simulated mixed infections by combining SA populations from different samples at 7:3 ratios. Detecting co-carriage requires sufficient polymorphism between the strains so that mixed allele frequencies can be detected, and the relative frequencies of each strain must be high enough to distinguish between normal population variations. In the easy examples, where there was an average of 79 (sd=1.16) polymorphic sites between the mixed strains, the assay detected on average 70% of them with minor allele frequencies ranging from 10 to 60% (Fig. 5 and Table S10). However, in one mixture (sample 3 at 70% and sample 12 at 30%), none of the expected polymorphisms were detected. This was likely due to a failure in the mixture preparation because the reciprocal mixture (sample 12 at 70% and sample 3 at 30%) successfully recovered the expected polymorphisms. In the more challenging cases, only 1–4 polymorphic sites differentiated the strains, and the assay detected 66% of them on average. The single polymorphism that was missed in the mixtures of samples 9 and 10 was due to failed amplification of the locus rather than a failure to detect the mixture of alleles. These results suggest that the AmpSeq assay can effectively identify co-carriage in cases where there is sufficient genetic variation between strains and an adequate relative abundance of both strains within the sample.

## Discussion

### Genomic resolution of AmpSeq assay

We have developed an AmpSeq assay targeting highly discriminatory regions of the SA core genome. These regions were selected based on their ability to capture phylogenetically informative SNPs while maintaining broad amplification across diverse SA genomes. Our results demonstrate that the phylogenetic tree generated from the AmpSeq assay approximates the topology of the core genome SNP tree. Despite the substantial difference in genomic coverage between WGS and AmpSeq (185,331 SNPs vs. 3,476 total genomic positions), the AmpSeq tree largely preserved the major phylogenetic groupings observed in the core genome SNP phylogeny, with sequence types clustering consistently. These findings suggest that our AmpSeq assay can provide effective strain differentiation with higher throughput and more cost-effectively than WGS.

Compared to MLST, our AmpSeq assay provides higher resolution and more closely aligns with the core genome SNP phylogeny. This improved resolution allows for better discrimination within sequence types, making it easier to differentiate closely related strains. Furthermore, traditional MLST schemes rely on long amplicons that may not be fully captured by short-read sequencing technologies, and protocols generally require separate amplification and sequencing of each target. In contrast, our AmpSeq assay is specifically designed for modern sequencing platforms with primers optimized for efficient multiplex amplification in a single reaction.

### Characterizing SA carriage in longitudinal study

This study was an initial test on a small subset of samples to establish the utility of our AmpSeq approach in characterizing SA carriage for large-scale studies. In our small subset of nine participants, we showed that they carried seven distinct sequence types (nine distinct AmpSeq genotypes) and that the colonizing strains were stable for up to 18 weeks. Participants who were colonized in both the nose and the mouth tended to carry the same strain at both body sites, and there were no cases of co-carriage of multiple strains. Although these results cannot be extrapolated for the whole long-term care facility population, they do demonstrate that this AmpSeq approach offers a cost-effective method of providing intermediate-level resolution for strain typing SA populations directly from samples without culture. When expanded to the analysis of our full large-scale study, this assay will allow us to understand population-level trends in carriage and facilitate high-powered analyses. It will enable us to characterize strain types circulating within the long-term care facilities, track how often strains change due to recolonization, estimate the rate of co-carriage and establish associations between strain types and demographic data.

### Detection of putative transmission between individuals

Our AmpSeq assay can rapidly eliminate putative transmission events by identifying cases where individuals do not share matching genotypes. While our method provides higher resolution than MLST at distinguishing sequence types, it does not offer the level of resolution required to fully confirm transmission events or determine directionality. For definitive verification, higher-resolution whole-genome or deep whole-population sequencing is required. We and others have developed such methods [40,71] to better characterize transmission dynamics. However, to efficiently identify potential transmission pairs, our AmpSeq assay can serve as an initial screening tool. By filtering out unlikely cases, we can reserve costly WGS or deep population sequencing for the most probable transmission events. In the samples tested here, our amplicon targets detected SNPs in strains between individuals that carried the same sequence types, and we could therefore rule out any transmission between individuals.

### Detection of putative host reservoirs and sources of spread to other body sites

The nares are commonly reported to be the most frequent site of colonization and are a likely reservoir for transfer to other body sites [2,11, 16, 19, 20, 72,74] and source of endogenous infections [72,73]. Our findings were consistent with this body of literature. In individuals with positive samples at both body sites, the nasal samples were typically positive more frequently than the oral samples. We observed that nasal samples consistently exhibited higher DNA quantities [16], and they carried more diversity; however, while these results were consistent, we cannot rule out the possibility that these differences may have been influenced by systematic variation in sample collection or differences in the microbial load relative to the community composition at each body site. Additionally, many low-frequency variants found in nasal samples were also detected in oral samples, but not vice versa, indicating that the nasal population may seed other body sites within the same host. The persistence of these low-frequency variants across multiple weeks further underscores the stability and central role of the nasal population in long-term carriage. While previous studies have identified the anterior nares as a major reservoir for SA, large-scale genotyping studies at multiple body sites have been limited due to the high cost of WGS and the low discriminatory power of MLST. Our high-throughput and cost-effective AmpSeq approach could enable more exhaustive sampling of body sites across large populations, providing a more detailed and statistically robust understanding of SA reservoirs.

### Caveats

One of the goals of this study was to understand the microvariation that exists within colonizing SA populations and utilize this information to gain insights into population dynamics, evolution and epidemiology. While PCR can amplify low-abundance sequences, it is also prone to introducing errors during amplification, which can create false-positive variants. This issue is particularly relevant when dealing with rare variants that occur at low frequencies, as distinguishing true biological variants from PCR- or sequencing-induced artefacts becomes difficult. Although we demonstrated that ~35% of rare variants detected in AmpSeq were also detected through parallel deep population WGS, our study did not include technical replicates or molecular barcoding techniques [75], which are typically used to identify spurious variants introduced during PCR amplification. Instead, we relied on the availability of multiple samples collected from the same individual across different time points to assess the persistence and reliability of rare variant detection. We found that many rare variants were consistently maintained across multiple samples for up to 18 weeks. The maintenance of low-frequency alleles (~0.3% on average for the nasal samples) for long periods of time suggests that selection on these loci may be weak and/or the effective size of the colonizing population is high enough that these variants are not rapidly fixed or removed through genetic drift. True technical replicates and/or molecular barcoding would be required to determine accurate time frames for shorter-lived true biological variants. But the fact that a non-trivial number of rare variants were maintained across all samples suggests that longer sampling intervals could be sufficient for tracking the dynamics of colonizing populations over time.

### Future applications

The approach described in this article has several potential applications for large-scale research studies, and we intend to continue employing this strategy as we systematically explore broader patterns of carriage and transmission. Our approach is ideal for (1) cost-effectively characterizing strain types carried by a large group of hosts and tracking changes in strain types over time, (2) identifying multi-strain infections – targeted AmpSeq provides deep coverage of multiple targets which can reveal mixtures of haplotypes within a population, (3) studies that require high statistical power, like associating strain types with certain host characteristics or body sites and (4) identifying reservoirs for spread and transmission – shared rare variants can help establish sources of spread between body sites and transmission to other individuals and help quantify how much diversity is transferred between body sites and between hosts. Because we can amplify directly from the collection media without culturing, this approach produces rapid results. While this method is not intended to produce definitive results regarding genotype similarity or validate transmission cases, it serves as an efficient and inexpensive screening tool to identify samples of interest for further validation. This can significantly reduce the need for WGS to only a subset of samples, thereby conserving resources while still obtaining high-resolution data.

## Conclusion

Our small-scale study demonstrates the utility of targeted AmpSeq for characterizing colonizing SA populations and detecting and tracking rare variants within SA populations. The findings provide valuable insights into the persistence of these strains and rare variants over time and offer practical guidance for future studies aimed at understanding bacterial diversity, sources of spread, transmission and infection control. As we continue to refine this approach, it holds promise for broad applications across pathogen species in both clinical and epidemiological research, ultimately contributing to more effective strategies for controlling the spread of bacterial pathogens.

## Supplementary material

10.1099/mgen.0.001427 Uncited Supplementary Material 1.

10.1099/mgen.0.001427 Uncited Supplementary Material 2.

## Abbreviations

AmpSeq: amplicon sequencing
MLST: Multilocus Sequence Typing
MRSA: methicillin-resistant SA
qPCR: Quantitative Polymerase Chain Reaction
RF: Robinson–Foulds
SA: *Staphylococcus aureus*
SNVs: single nucleotide variants
WGS: whole-genome sequencing
