# Supplementary material for: High-throughput targeted amplicon screening tool for characterizing intrahost diversity in Staphylococcus aureus directly from sample
Source: Microb Genom. 2025 Jun 25;11(6):001427. doi: 10.1099/mgen.0.001427 (PMC12198441; doi:10.1099/mgen.0.001427)
Supplement: Uncited Supplementary Material 1. [file mgen-11-01427-s001.pdf]

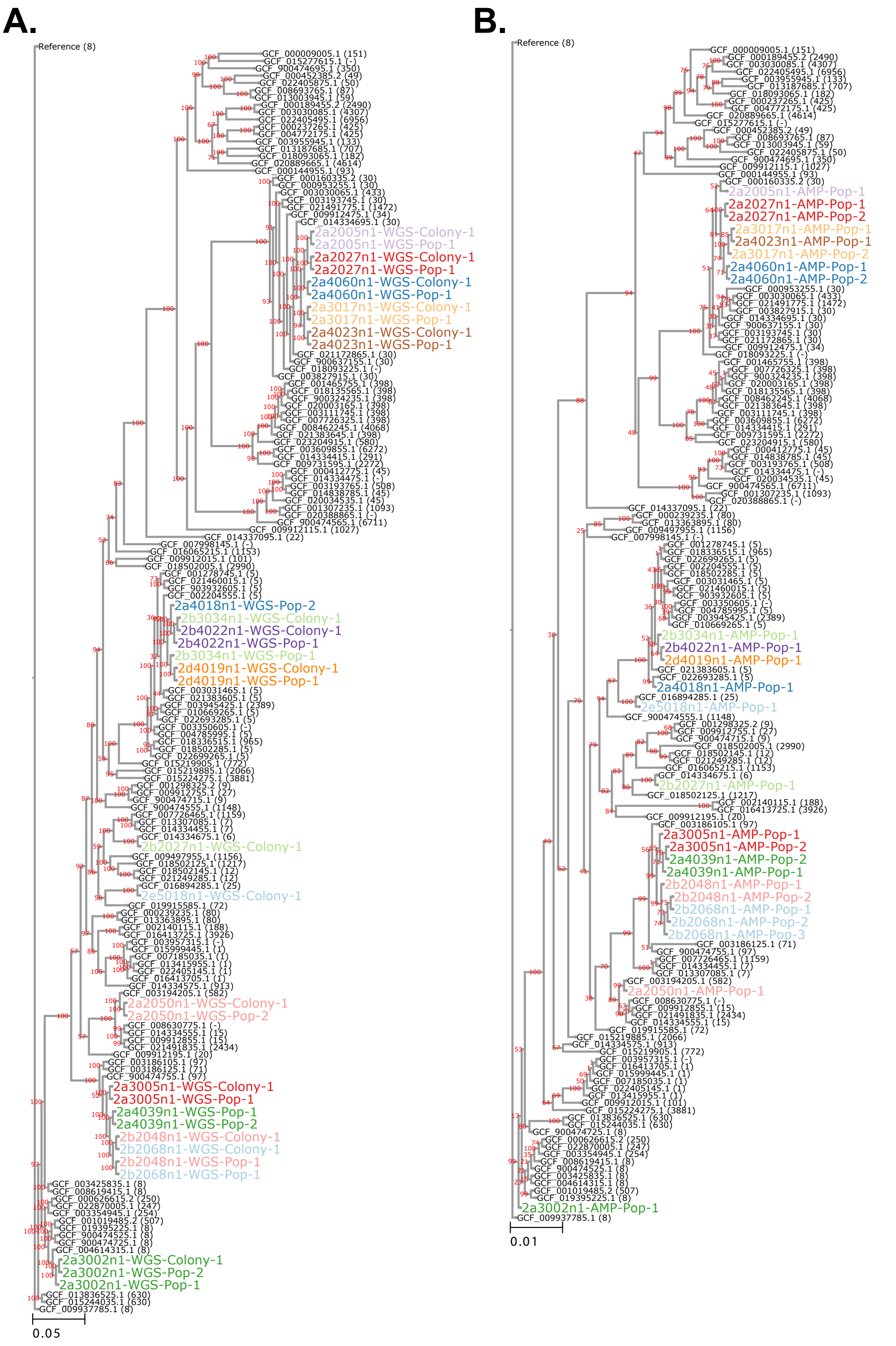

Supplemental Figure 1. Maximum likelihood phylogeny showing the placement of nasal samples collected from 17 individuals among *Staphylococcus aureus* reference genomes.

Phylogenetic relationships were inferred using core genome SNPs from WGS (A) and amplicon sequences from the AmpSeq assay (B). Study samples are labeled in color, while reference genomes are labeled in black, with the sequence type in paratheses. The first part of each sample label corresponds to the ID for the individual. Samples obtained from a single colony are labeled 'Colony' and those from a pooled population of colonies are labelled "Pop". The final number in the label denotes the technical replicates. Branch support values are indicated in red.

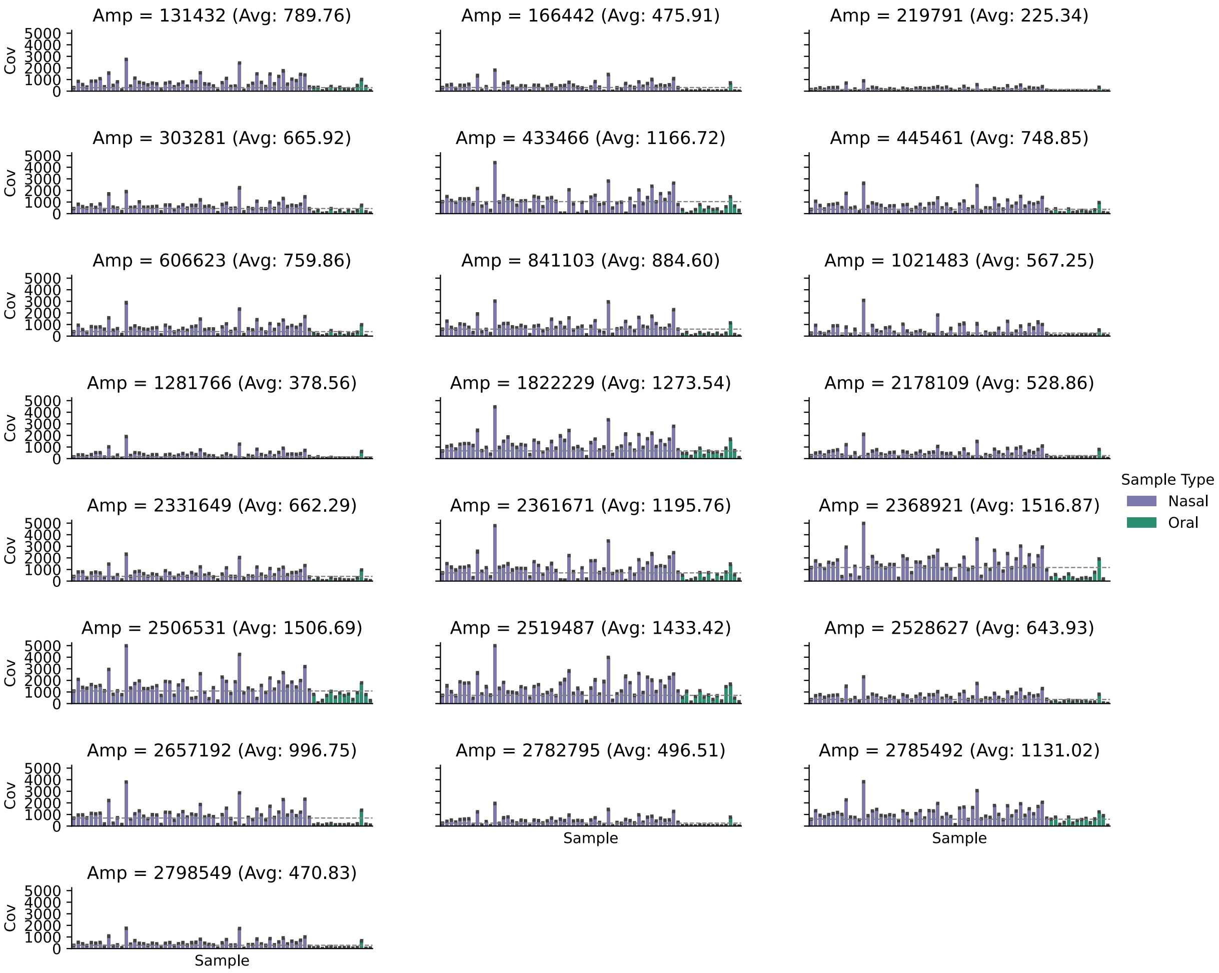

Supplemental Figure 2. Consistent coverage was achieved at 22 of the amplicon targets. The figure shows the average coverage (error bars indicate standard deviation) for each sample for each site within the amplicon targets. The average depth of coverage across all samples was 716 (sd=492). Each amplicon had at least 50/69 samples with greater than 50x coverage. The minimum average coverage for an amplicon was 255x for amplicon 219791. The coverage for the nasal samples (purple) was generally higher (mean=827, sd=480) than the oral samples (green, mean=279, sd=232).
